# Cerebral Visual Impairment Characterized by Abnormal Visual Orienting Behavior With Preserved Visual Cortical Activation

**DOI:** 10.1167/iovs.62.6.15

**Published:** 2021-05-13

**Authors:** John P. Kelly, James O. Phillips, Russell P. Saneto, Hedieh Khalatbari, Andrew Poliakov, Kristina Tarczy-Hornoch, Avery H. Weiss

**Affiliations:** 1 Roger H. Johnson Vision Clinic, Seattle Children's Hospital, Division of Ophthalmology, Seattle, Washington, United States; 2 University of Washington, Department of Ophthalmology, Seattle, Washington, United States; 3 University of Washington School of Medicine, Department of Otolaryngology, Seattle, Washington, United States; 4 Seattle Children's Hospital, Department of Neurology/Division of Pediatric Neurology, Neuroscience Institute, Seattle, Washington, United States; 5 Seattle Children's Hospital, Department of Radiology, Seattle, Washington, United States

**Keywords:** visual evoked potentials (VEPs), cerebral visual impairment (CVI), neuroimaging, eye movements

## Abstract

**Purpose:**

Children with cerebral visual impairment (CVI) often have abnormal visual orienting behaviors due to impaired or damaged visual cortex. Alternatively, visual-cortical function is intact but visual information is not transformed downstream into an appropriate oculomotor output (visuomotor dysfunction). We examined visual, anatomic, and oculomotor assessments to distinguish visuomotor dysfunction from CVI associated with severely reduced visual-cortical response.

**Methods:**

We reviewed the medical records from children with CVI having abnormal visual orienting behaviors, normal ocular examinations, and born near term. Relevant data were visual evoked potentials (VEPs), Teller card acuity, eye movements recorded by video-oculography (VOG), and neuroimaging (magnetic resonance imaging [MRI]) including diffusion tensor imaging (DTI) tractography.

**Results:**

Thirty subjects had visuomotor dysfunction based on a normal VEP; of these 33% had a normal MRI and 67% had white matter abnormalities associated with metabolic disease and/or decreased volume of brain parenchyma. VOG recordings showed smooth pursuit gains were uniformly reduced and saccades were dysmetric but followed the main sequence. Ten subjects had severe CVI based on VEPs at noise levels; visual acuities and MRI findings overlapped those of the visuomotor dysfunction group. Developmental delay, seizures, microcephaly, and hypotonia were common across all groups. All subjects with an abnormal conventional MRI had abnormal metrics on DTI tractography from the occipital lobe.

**Conclusions:**

A subset of patients with CVI have abnormal visual orienting behaviors despite a normal VEP (visuomotor dysfunction). A majority have abnormal white matter metrics on tractography suggesting a downstream defect in sensorimotor transformation. Clinically, visuomotor dysfunction is indistinguishable from severe CVI.

Cerebral visual impairment (CVI) is a frequent cause of vision loss in children born in developed countries.^1^ CVI is a complex disorder that can be broadly defined as impaired visual orienting behaviors with evidence of a cortical deficit that cannot be accounted for by ocular pathology or an oculomotor disorder.^1^^,^^2^ Common causes of CVI include hypoxic/ischemic injury or cerebral infarct (often in the setting of prematurity), hydrocephalus, meningitis, and cortical malformations.^1^^–^^6^ CVI is frequently associated with other neurological deficits, such as cognitive delay, cerebral palsy, and seizures. Magnetic resonance imaging (MRI) helps to localize and define the underlying brain pathology in CVI. Because most children who present with CVI cannot communicate verbally or are developmentally delayed, visual acuity can be assessed by preferential-looking tests (e.g. Teller acuity cards) or by visual evoked potentials (VEPs). The VEPs provide objective assessment of visual-cortical function in children with CVI.^2^^,^^3^^,^^7^ The VEP reflects population activity in both visual striate and visual association cortices^8^^–^^10^ predominately from central 15 degrees of the visual field.^11^^,^^12^

Abnormal visual orienting behaviors in CVI are often attributed to reduced vision from damage or dysfunction of striate and extra-striate cortex. In this case, spatial and temporal information from visual cortex is too degraded for planning and execution of precise visually guided eye movements. Alternatively, striate and extra-striate cortex could be functionally intact but transmission of visual sensory information to downstream visual cortical and oculomotor areas is diminished or completely interrupted. Specifically, there can be an anatomic or functional disconnection to parietal cortex, frontal cortex, the cerebellum, vermis, midbrain, and brainstem. Given an inability to generate appropriate visually guided behaviors, visual acuity using preferential-looking acuity cards may be difficult to interpret because the test requires reliable gaze shifts.^1^ For example, some children with CVI, who were initially diagnosed as being visually impaired on preferential-looking acuity cards, were eventually found to have normal visual acuity after repetitive testing.^13^^,^^14^ Therefore, the VEP can be critical for assessment of vision in these children if there is a defect in sensorimotor transformation into visually guided behaviors.

Evidence that some children with CVI have a defect in sensorimotor transformation to downstream oculomotor areas have been published in several case studies.^15^^–^^17^ Bodis-Wollner et al.^15^ reported a “blind” child that had a normal VEP response but damage to extra-striate areas in the occipital lobe. The abnormal behaviors were attributed to disconnection between the striate visual cortex and the downstream cortical areas. Wygnanski-Jaffe et al.^16^ reported a subset of children with CVI having robust responses on VEPs despite appearing “clinically blind.” These authors noted several of their subjects had delayed myelinization or volume loss of deep white matter. Previously, we described a group of infants with extensive neuronal migration defects beyond visual cortex (polymicrogyria) having robust VEPs despite subnormal visual tracking behaviors.^17^ In further support of defects in sensorimotor transformation, there are case reports of premature children with CVI showing abnormal white matter tractography from visual cortex to dorsal and ventral processing streams on diffusion-tensor imaging (DTI).^18^^–^^20^

The purpose of this study was to characterize children with CVI in which visual-cortical function is intact but visual information is not transformed into an appropriate oculomotor output (“visuomotor-dysfunction”). We hypothesized there would be consistent disorders across eye movement studies (saccadic, smooth pursuit, optokinetic nystagmus [OKN], and gaze holding) in those with visuomotor dysfunction. We also searched for abnormal white matter tracts connected with striate cortex. To address these hypotheses, we reviewed cortical function assessed by VEPs, preferential-looking visual acuity, ophthalmological clinical findings, oculomotor recordings, and white matter metrics using DTI tractography. Because abnormal visual orienting behaviors in CVI should be distinguished from that caused by visual cortical dysfunction, we compared the visuomotor-dysfunction group to children with CVI having DTI imaging and severely reduced VEPs (“severe CVI”). Confounding factors were reduced by excluding subjects with complications of prematurity, cortical migration defects, or delayed visual maturation.^3^^,^^21^

## Methods

The research was conducted in compliance with accredited institutional review board approval. Patient records from Seattle Children's Hospital were retrospectively reviewed. All subjects were referred to Seattle Children's Hospital Ophthalmology Clinic from a wide range of outside medical specialists for concerns of reduced vision, lack of visual orienting, or poor tracking to high contrast toys, with or without associated neurological deficits. Inclusion criteria for the visuomotor dysfunction group were (1) clinically abnormal visual tracking/orienting behaviors to targets at near or distance (see the eye movement section below) that could not be explained by ocular motor apraxia or other motility disorders; (2) a VEP in the normal range confirmed by objective analysis of electroencephalogram (EEG) epochs; (3) normal dilated fundus examination; (4) gestational age ≥ 34 weeks; (5) neuroimaging by MRI without evidence of intraventricular hemorrhage, hypoxia-ischemia, cerebral infarct, traumatic brain injury, hydrocephalus, or significant cortical migration defect (e.g. pachygyria, polymicrogyria, and schizencephaly); and (6) developmental assessment by a pediatrician. Results of genetic and metabolic testing were recorded for subjects subsequently found to have white matter changes in the presence of metabolic disease or deficiencies of mitochondrial electron transport (defined as 20% of control values).^22^ Inclusion criteria for the severe CVI group was a VEP waveform and signal at background noise levels (see the VEP section below) but required DTI studies, and did not exclude hypoxic-ischemic injury.

All eye examinations were performed at Seattle Children's Hospital that included external examination, motility, and dilated fundus examination. Preferential-looking assessments by Teller Acuity Cards were performed by trained observers in the Ophthalmology Clinic. To standardize visual acuity measured at different ages, best corrected visual acuity was converted to age-corrected log minimum angle of resolution (logMAR).^23^ The logMAR value represents the logarithmic deficit relative to the average normal acuity for the equivalent age. For example, if the normal average was 3.1 cycles/degree, a subject with 1.6 cycles/degree acuity of the same age would have an age corrected logMAR value of 0.3. Another child of the same age with 0.31 cycles/degree acuity would have an age corrected logMAR value of 1.0, and so forth. Reporting values in age-corrected logMAR is more accurate than Snellen optotype acuity (the latter is not always equivalent) and indicates the amount of visual-acuity deficit compared to normative values for age.

All subjects had clinical assessment of eye movements. The spectrum of abnormal visual tracking/orienting behaviors was comparable to that described by Jan et al.^14^ In addition to assessing versions, the vestibulo-ocular reflex could be examined qualitatively to confirm the oculomotor system was intact. Our specific criteria were one or more of following: (1) gaze holding was defined as abnormal if nystagmus was present; (2) smooth pursuit was abnormal if there was an absence (saccadic tracking or no response) or a reduction (reduced gain) in smooth eye movement in response to a sinusoidally moving target; (3) saccades were abnormal if they were absent, dysmetric (hypo- or hyper-metric), slow, or had abnormal latency in response to sequentially presented targets; and (4) OKN was abnormal if the response was absent, nystagmus was poorly formed, or the gain was abnormal. When possible, eye movements were recorded using binocular infrared video-oculography (Sensorimotoric Instruments, Berlin) as previously described.^24^ The subject viewed a 60 degrees projection screen at 80 cm. Saccades were elicited by random 5 degrees to 20 degrees horizontal target steps, and smooth pursuit by sinusoidal target drift ±10 degrees along the horizontal meridian at peak velocities of 10, 20, and 30 degrees/sec. OKN was elicited by square wave gratings (0.1 cycles/degree; 80% contrast) drifting horizontally or vertically at constant velocities of 15, 30, and 45 degrees/sec. Eye movements were analyzed using custom software (http://faculty.washington.edu/jokelly/voganalysis).

VEP recordings followed published procedures^25^ using International Society for Clinical Electrophysiology of Vision (ISCEV) recommendations.^12^ The reference was at Cz, ground at Pz, and an active electrode at Oz. Additional locations at either O1, O2, T5, T6, Inion, and POz were used to analyze hemispheric symmetry or EEG artifacts and were not reported. Subjects viewed a raster monitor (Eizo TX-C7, 22 × 17 degrees, 100 Hz frame rate) in darkness. Patterns were reversing-checks (163, 84, 18, and 9 arc minutes, 80% contrast, 2.74 reversals per second) and onset-offset of sinewave grating onset (0.5 cycles/degree; 80% contrast; on for 150 ms then off for 500 ms at constant mean luminance). However, inadequate fixation limited VEP data analysis to 163 arc minute checks and 0.5 cycles/degree grating across all subjects. Additional check sizes for extraction of grating acuity^26^ were available in a subset of subjects but only used to confirm detectable responses to smaller check sizes. Fixation was aided by a small toy at the center of the display and VEPs were recorded during an alert state. The recording was paused when fixation (by corneal light reflex) was not directed to the stimulus. Each time the recording was resumed, an additional trial was added for averaging. There was a minimum of 40 stimulus presentations per stimulus, which could be doubled if the child was inattentive.

Given the abnormal visual behaviors and seizures in these subjects, specialized averaging methods were used. For each stimulus, EEG epochs were overlaid on top of each other. Epochs with spikes or large background noise were individually removed by visual inspection. The Oz location then underwent “standard” averaging. The same epochs then underwent discrete Fourier transforms for “FT-selective” averaging.^25^ FT-selective averaging also assumes a stationary and deterministic VEP signal (having constant amplitude and latency throughout the averaging process). In contrast, eye movement artifacts and abnormal background EEG generate noise variations in both amplitude and phase in the Fourier transform.^25^^,^^27^ Epochs were converted into magnitude and phase plots at temporal frequencies from 5.5 to 21.9 Hz. Then the signal at each frequency was taken as the ratio of its magnitude to the 95% confidence circles in the phase plot. A signal-to-noise ratio (SNR) was defined as the sum of all ratios across all temporal frequencies. The algorithm then reiteratively selects EEG epochs with consistent amplitudes and phases (lower 95% confidence circles) until there is no improvement in SNR. A large SNR indicates EEG epochs have large amplitude components with accurate phase-locking to the stimulus presentation. After the reiteration, the accepted epochs are averaged in the time domain to generate the FT-selective VEP. If the SNR was ≤ 1.3 after FT-selective averaging, then the signal was not statistically different from background noise,^25^ in which case, the algorithm lacks a phase-locking signal and outputs the standard average. After each averaging method, VEPs were digitally filtered 1.5 to 41.3 Hz, then scored for latency (time to the prominent positive peak after 80 ms) and amplitude (voltage difference between this peak and the preceding negative deflection if present, or baseline). Control data were taken from published data using an identical analysis.^25^

The MRI protocol included high-resolution sagittal T1 magnetization-prepared rapid acquisition of gradient echo (MPRAGE; repetition time [TR] ms = 1450–1950; echo time [TE] ms = 2; field of view (FOV) mm, 190–256), axial and coronal T2-weighted spin-echo (TR = 3200–5720 ms; TE = 75–386 ms; FOV 146–250), axial fluid‐attenuated inversion recovery (FLAIR). All MRIs were reviewed by a pediatric neuroradiologist.

DTI scans were acquired on Trio or Prisma scanners at 3 Tesla (Siemens, Erlangen, Germany) using a single-shot echo planar imaging sequence (TR = 4300–6813 ms; TE = 65–96 ms; and FOV = 190–230 mm). The b-values for DTI were 0 and 1000 s/mm^2^. Two DTI acquisition schemes occurred during the study period: (1) a sequence of 30 gradient-encoding directions with in-plane resolution of 2 mm and slice thickness of 2.2 mm (matrix size 112 × 112) with 55 to 65 sections covering the entire brain; (2) a sequence of 10 gradient-encoding sampling directions with in-plane resolution of 1.8 mm and slice thickness of 3.3 to 3.5 mm (matrix size 128 × 128) with 34 to 50 sections covering the entire brain. DTI data and deterministic fiber-tractography were analyzed using DSI Studio (http://dsi-studio.labsolver.org), which showed good validation with weak MR signals.^28^^,^^29^ Scans had to meet quality criteria of DSI Studio. Tracking parameters were default anisotropy threshold, 60 degrees angular threshold, 0.1 mm step size, track length between 30 and 400 mm, and a maximum of 10,000 seeds. DSI Studio performed automatic seeding of white matter tracts based on the HCP842 tractography atlas with an internal fractional anisotropy threshold. The software used nonlinear registration of subject data to MNI space, then placed seeds within the HCP842 tract volume. The generated streamlines were compared to each associated fiber tract from the HCP842 atlas using Hausdorff distances. Streamlines were retained only if they matched the atlas target track. If the automated tracking method found less than 30 fiber tracts, the region was manually re-seeded, and the anisotropy threshold set at 0.17 based on pediatric subjects with abnormal white matter.^30^ Fiber tracking was chosen for projections to and from the occipital lobe that included the optic radiations (ORs), inferior fronto-occipital fasciculus (IFOF), superior longitudinal fasciculus (SLF), vertical occipital fasciculus (VOF), and corpus callosum (CC). Additional tracks included the cortico-pontine tracts; fronto-pontine (FP), occipito-pontine (OP), parieto-pontine (PP), temporo-pontine (TP), and white matter of the cerebellum (Cbl) and vermis. Fiber tracts or U-shaped fibers were removed if clearly aberrant. For isolation of the OR, we removed fibers anterior to the lateral geniculate body and fiber crossing regions near Meyer's loop. Fibers of the splenium were only included in the reconstruction of the CC. The CC, OP, TP, and PP tracts were reviewed to exclude fibers from the corticospinal tract, which appear as long tracts vertically extending from the corona radiata and past the pons. We report the average fractional anisotropy (FA), radial diffusivity (RD), and mean diffusivity (MD) of bi-hemispheric reconstructed tracts. DTI metrics were compared to VEPs if both tests were performed within 1 month for subjects 4 to 6 months old, or within 1 year for subjects > 6 months (ranges when VEPs are stable with age).^25^^,^^31^

DTI imaging controls were matched for age, DTI protocol, and acquisition date within 6 months, from a database of children without visual disorders, with normal neuroimaging (T1-MPRAGE, T2, T2/FLAIR, and ADC) done for isolated seizures, developmental delay, Rolandic epilepsy, unexplained isolated events, or migraine.

## Results

Out of 550 records, 40 subjects met the inclusion criteria (16 boys; mean age at first visit = 1.4 years, median = 0.9 years of age). All subjects were nonverbal at the time of VEP testing. Visual acuity, MRI, and laboratory findings are listed in Supplementary Table S1. Thirty subjects were classified as having visuomotor dysfunction based on VEP amplitude, latency, and SNR in the range of controls (after FT-selective averaging). The remaining 10 subjects classified as having severe CVI based on a VEP waveform and SNR at background noise levels. The severe CVI group served as a comparison group only (Table). The visuomotor dysfunction group was further separated into three groups. The first group (*n* = 10) had a normal MRI (“normal MRI”). The second group (*n* = 14) had an abnormal conventional MRI (“abnormal MRI”) predominated by bi-hemispheric abnormality of the white matter (delayed myelination, abnormal signal, and thinning of the corpus callosum). The third group (*n* = 6) had low-normal VEP amplitudes with an inborn error of metabolism also with predominately white matter signal abnormalities on MRI (“metabolic disorder”). The spectrum of MRI abnormalities in the severe CVI group overlapped that of the visuomotor dysfunction groups. Almost all subjects in each group had developmental delay in gross motor and cognitive skills. A majority of subjects had hypotonia; seven had spasticity or hypertonia, and three had dystonia. Genetic testing or biochemical assay in 25 subjects revealed a heterogenous range of chromosomal, genetic, or metabolic disorders (Supplementary Table S1). Seven subjects had genetic mutations associated with infantile epilepsy.

**Table.** Summary Data

	VMD With Normal MRI	VMD With Abnormal MRI	VMD with Metabolic Disorder	Severe CVI	All Controls
***N***	10	14	6	10	26
**Mean age**	2.2 (1.6)	1.5 (1.1)	2.5 (3.7)	1.1 (0.6)	2.1 (1.5)
**logMAR**	1.9	1.9	1.4	2.6	
**Range**	3.1 to < 0.23 cy/deg	6.5 to < 0.23 cy/deg	9.8 to < 0.23 cy/deg	1.3 to < 0.23 cy/deg	
**Stable gaze**	100%	86%	50%	50%	
**Strabismus**	40%	57%	67%	50%	
**Microcephaly**	20%	36%	50%	50%	
**DD**	90%	93%	100%	100%	
**Hypotonia**	50%	93%	100%	70%	
**Seizures**	50%	64%	33%	80%	
**Check VEP****^†^**	**Mean (SD)**	**Mean (SD)**	**Mean (SD)**	**Mean (SD)**	**Mean (SD)**
**Amplitude**	38.0 (20.4)	38.2 (14.9)	22.5 (7.7)	5.0 (2.9)^***^	36.6 (11.2)
**SNR**	72.4 (26.1)	73.6 (20.3)	68.0 (6.2)	1.2 (0.6)^***^	112.1 (61.6)
**Latency**	98.0 (8.3)	114.4 (27.4)	109.3 (16.6)	126.4 (31.2)	102.9 (7.2)
**Onset VEP****^†^**					
**Amplitude**	43.9 (26.4)	34.8 (24.8)	29.3 (11.3)	10.9 (13.4)^**^	33.8 (11.0)
**SNR**	67.8 (19.0)	54.6 (23.6)	63.3 (21.0)	12.3 (24.0)^***^	70.1 (30.6)
**Latency**	115.0 (30.9)	130.5 (61.7)	103.8 (11.8)	130.3 (35.3)	100.4 (11.6)

Age in years; logMAR visual acuity corrected for age; cy/deg is grating acuity in cycles/degree; microcephaly is < 5% of normative data for occipital frontal circumference.

VMD = visuomotor dysfunction; CVI = cortical visual impairment; DD = developmental delay.

† Averaging using the FT-selective method; *P* values statistically significant from controls on *t*-test after Bonferroni correction.

^**^ = *P* < 0.001.

^***^ = *P* < 0.0001.

The Table summarizes clinical findings, visual acuity, and VEP data for the four groups. All logMAR acuities represent age-corrected values. At presentation, 22 of the 40 subjects had no response to the low vision Teller card (worse than 0.23 cycles/degree) and were assigned a logMAR value of 3.0. Differences in logMAR were not statistically significant between groups (*P* > 0.25, Mann Whitney test; *P* = 0.42, Kruskal Wallis test). Follow-up visual acuity was available in 19 of the visuomotor dysfunction subjects. For these subjects, logMAR averaged 2.0 at presentation and averaged 1.3 at their last visit after a mean follow-up of 2.7 years. Follow-up visual acuity was available in 11 subjects who had measurable acuity at presentation. Of these subjects, logMAR averaged 0.84 at presentation and averaged 0.76 at their last visit after a mean follow-up of 2.5 years. Despite abnormal eye movements (lack of visual orienting / tracking, random conjugate saccades, hypometric saccades, and absence of smooth pursuits), four subjects were found to have mild acuity deficits for age (logMAR <0.4) based on gross orienting responses using Teller cards (actual value worse than 9.8 cycles/degree).

### Oculomotor Data

**Figure 1.**
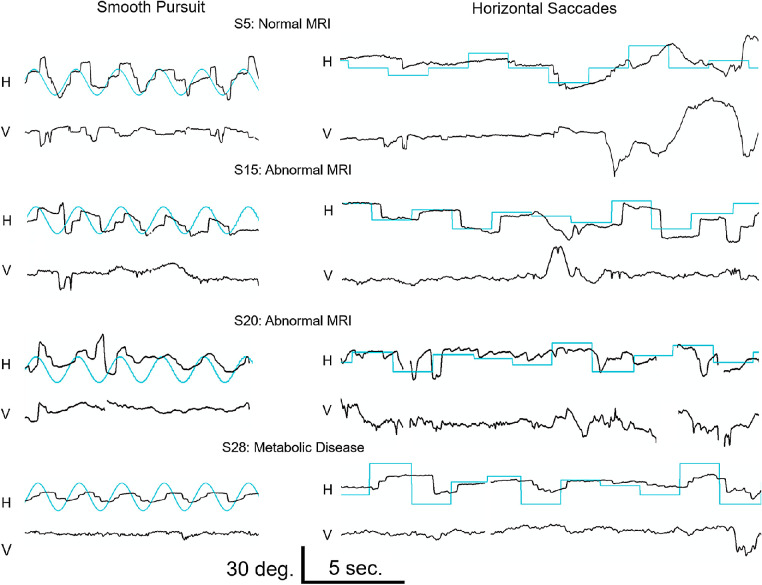
Sample eye movement recordings in four subjects (#5 with a normal MRI, #15 with delayed myelination, #20 with delayed myelination and partial agenesis of the corpus callosum, #28 with delayed myelination and respiratory complex I, IV deficiency; *top to bottom*). The *left side* shows smooth pursuit task of a target sinusoidally moving at 30 degrees/s. Horizontal (H) and vertical (V) eye position traces are shown with the target position (*blue traces*). The right side shows targeted saccade. Both the horizontal (H) and vertical (V) eye position traces are shown with the target position (*blue traces*).

Gaze-holding was mostly stable across subjects. For others, gaze-holding was either nontargeted roving eye movements, conjugate jerk nystagmus, or rotary nystagmus of < 10 degrees amplitude (Table). None had characteristics of congenital oculomotor apraxia. Strabismus was frequent in all groups. Due to technical considerations and cooperation, assessments of eye movements by video-oculography were limited to 11 subjects (*n* = 11 for OKN, *n* = 10 for horizontal saccades, and *n* = 8 for smooth pursuit), all of whom were in the visuomotor dysfunction group. Figure 1 shows eye movement data from 4 subjects. For horizontal smooth pursuit, subjects had saccadic tracking that was primarily hypometric. One subject (#20 with delayed myelination) responded with a brief period of a targeted hypometric saccade and low gain smooth pursuit. Average horizontal smooth pursuit gain across subjects was 0.28, 0.22, and 0.17 for velocities of 10, 20, and 30 degrees/s, respectively (<5% of our controls^24^). Subjects also had large vertical components not seen in our control children on horizontal tasks. There were no significant differences in smooth pursuit gain between subjects with or without MRI abnormalities.

**Figure 2.**
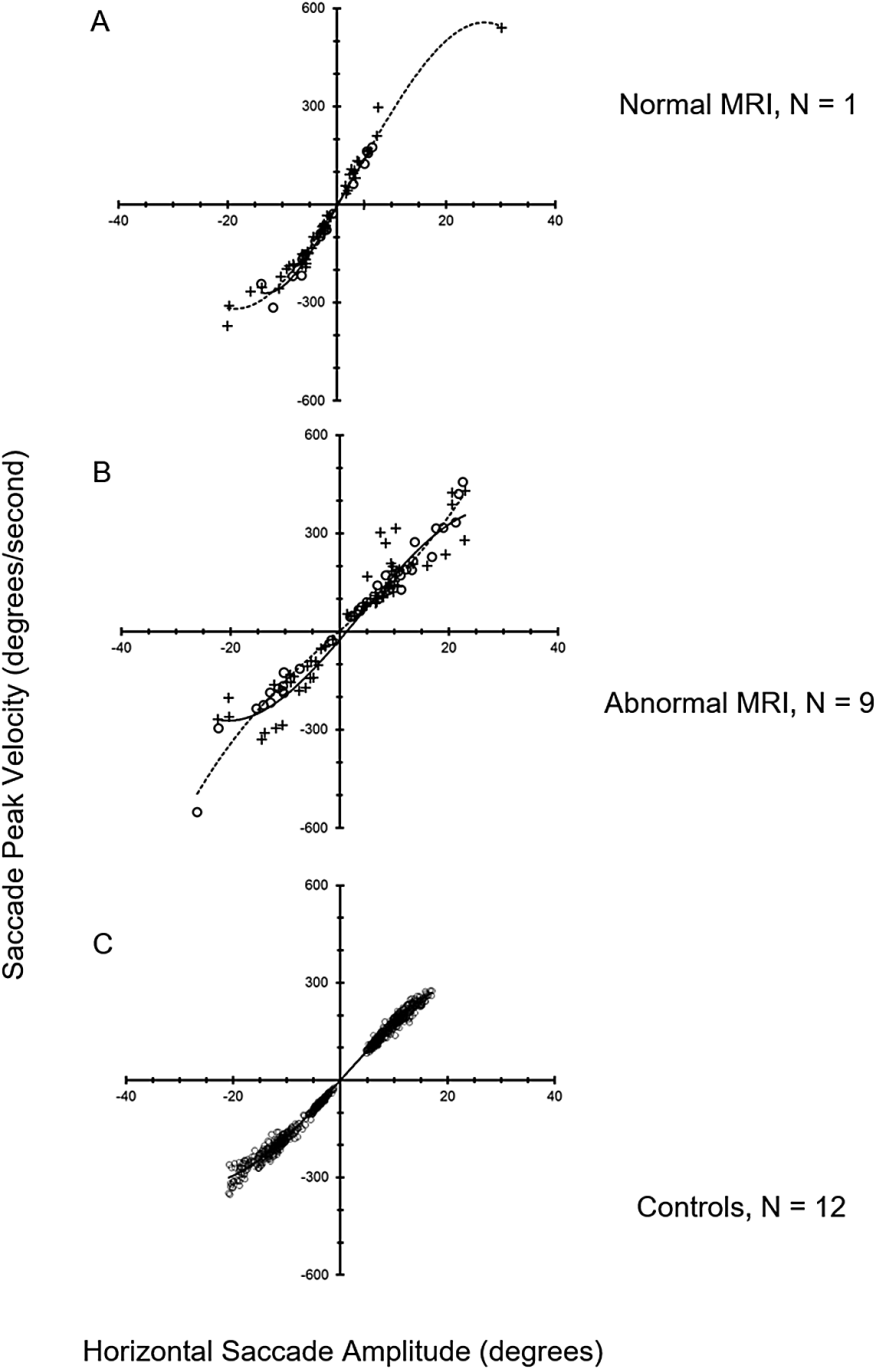
The relationship between saccade peak velocity and saccade amplitude (main sequence) for horizontal saccades. (**A**) A subject with a normal MRI, (**B**) 9 subjects with an abnormal MRI, and (C) 12 control subjects. *Open circles* and *solid line* fits are for saccades generated in the correct direction. *Crosses* and *dotted line* fits are for nontargeted saccades generated in the incorrect direction. The *lines* are third-order polynomial fits.

Saccades elicited to horizontally stepped targets were highly variable (see Fig. 1). Subject 5 (top row) intermittently generated hypometric saccades to leftward target steps with prolonged saccade latency. Subject 15 generated normometric, horizontal saccades with prolonged latency. Subject 20 showed only one hypometric saccade in response to a stepped target. Subject 28 generated multiple hypometric saccades in response to target steps but failed to acquire the target. Analyzing all saccades, regardless whether they were generated in the appropriate direction, subjects showed a systematic relationship between peak velocity and saccade amplitude (i.e. main sequence). Despite the uniform dysmetria across trials, there was a systematic main sequence across subjects (Fig. 2).

Horizontal and vertical OKN gains varied widely across subjects (Supplementary Fig. S1). For horizontal drifting gratings, most subjects had reduced OKN gain relative to controls for both rightward and leftward directions, especially at higher velocities. There were no consistent differences in OKN gains between subject groups.

### Visual Evoked Potentials

**Figure 3.**
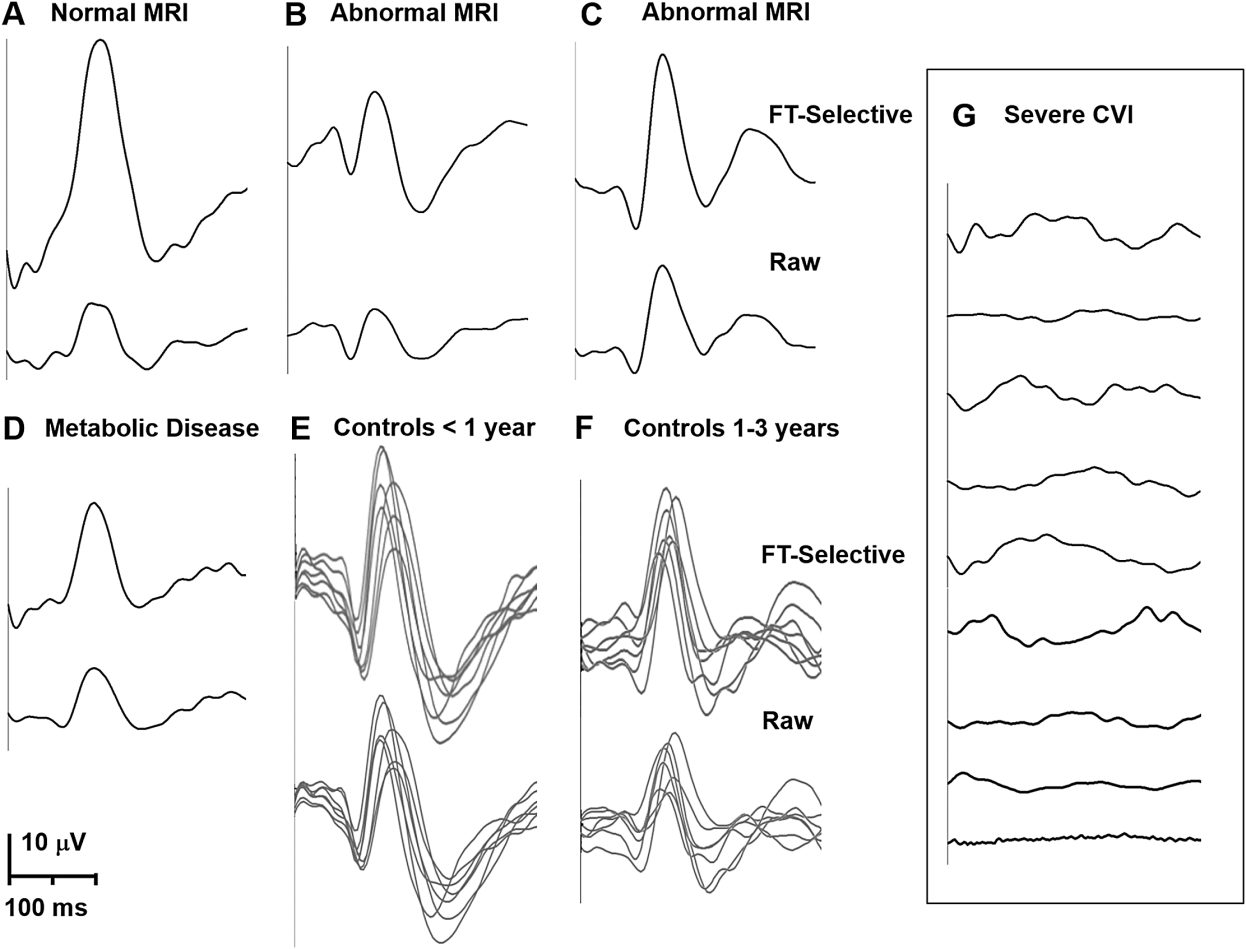
Sample visual evoked potential (VEP) waveforms compared to age-appropriate controls. Stimuli were reversing checkerboards. For each panel, the upper trace shows the VEP after FT-selective averaging while the lower trace is the raw average. The same subjects shown in Figure 1 are shown in this figure. (**A**) Waveforms from the subject with a normal MRI. (**B,** **C**) Waveforms from two subjects with abnormal MRI. (**D**) Waveforms from the subject with complex I, IV deficiency. (**E**) Sample waveforms from 7 controls of 0.3 to 0.9 years age. (**F**) Waveforms from 7 controls of 1 to 3 years age. (**G**) Range of waveforms from subjects with severe CVI.

Thirty-six controls were selected to match the age range of each group (*t*-test for age differences; *P* > 0.83). Figure 3 plots representative VEP waveforms, including the same subjects shown in Figure 1 (VEPs recorded 3–6 months before eye movements). Figure 3 demonstrates the VEPs in the visuomotor dysfunction were similar to controls despite abnormal visual acuity and oculomotor behaviors. FT-selective averaging generated larger responses without altering peak latencies (paired traces in Fig. 3).

Inspection of each subject's raw EEG epochs confirmed the FT-selective algorithm extracted epochs with improved phase-locking to the stimulus compared to standard averaging. Compared to standard averaging, FT-selective averaging increased VEP amplitudes by a factor of 1.6 (SD = 0.46, *P* < 0.0001) in controls and by a factor of 3.4 (SD = 2.36, *P* < 0.0001) in visuomotor dysfunction subjects. SNR was also significantly increased after FT-selective averaging in controls (factor of 1.7, SD = 0.59, *P* < 0.0001) and in visuomotor dysfunction subjects (factor of 3.3, SD = 1.7, *P* < 0.0001). Latency was not affected by FT-selective averaging (difference for controls = −0.7 ms, *P* = 0.53; difference for visuomotor dysfunction subjects = 1.88 ms, *P* = 0.29). Amplitude, latency, and SNR were not correlated with the number or percentage of epochs accepted after the FT-selective averaging technique (r^2^ < 0.11 for all correlations). The Table shows controls and visuomotor dysfunction subjects were not significantly different from each other after FT-selective averaging. The severe CVI group had significantly reduced VEP amplitude and SNR compared to controls, but latencies were not statistically longer. Of note, only standard averaging was used for the severe CVI group as there was no detectable signal for the FT-selective averaging algorithm.

### Neuroimaging

Abnormalities on conventional MRI were common, yet nonspecific among groups. The dominant finding was abnormal white matter (abnormal signal, delayed myelination, or reduction in white matter volume) in 23 of 40 subjects. Fourteen subjects had evidence of cerebral volume loss, and six subjects with decreased volume of the cerebellum. Six subjects showed either full or partial maturation of their delayed myelination on a follow-up MRI study (2 had a metabolic disorder). Four subjects had mega-cisterna magna. One subject had abnormal signal in the midbrain, and one subject had brainstem atrophy. Only one subject in the severe CVI group had hypoxic ischemic injury.

### Diffusion Tensor Imaging

**Figure 4.**
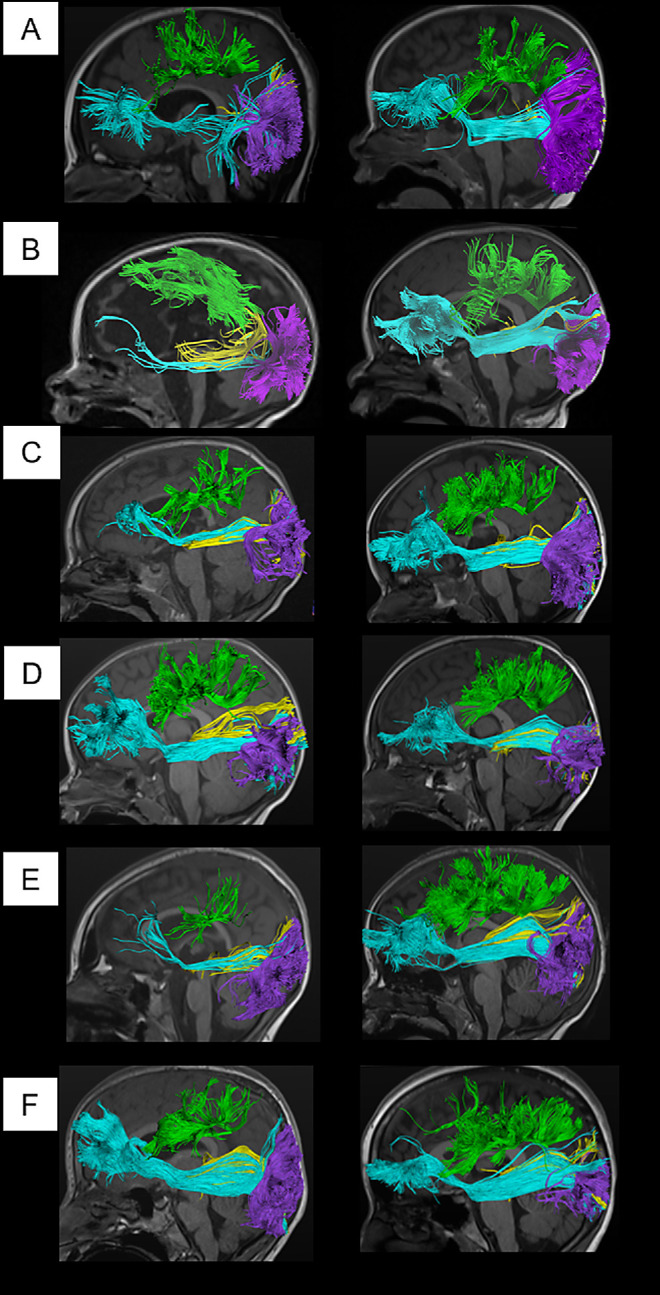
Reconstruction of four major white matter tracts in six subjects with visuomotor dysfunction (*left side*) paired with an age-matched control under the same imaging sequence (*right side*). Tracts shown are optic radiations in *yellow*, vertical occipital fasciculus in *purple*, superior longitudinal fasciculus in *green*, and inferior fronto-occipital fasciculus in *cyan*. (**A–F**) Show data from subject numbers 15, 25, 6, 4, 12, and 28, ordered in age from 0.5 years (*top*) to 14 years (*bottom*).

Adequate DTI data were obtained in 28 study subjects (*n* = 8 with normal MRI, *n* = 6 with abnormal MRI, *n* = 5 with metabolic disease, and *n* = 9 with severe CVI). White-matter tracts could be reconstructed in 555 of a total 560 tracts. One subject with severe CVI had no detectable FP or IFOF tracts bilaterally. Another subject with metabolic disease had no detectable right OP tract. Control DTI and tractography analyses consisted of a database of 43 separate children. Reconstructed tracts (Fig. 4) in visuomotor dysfunction subjects had similar shape and location to controls, but some subjects showed reduced volume.

**Figure 5.**
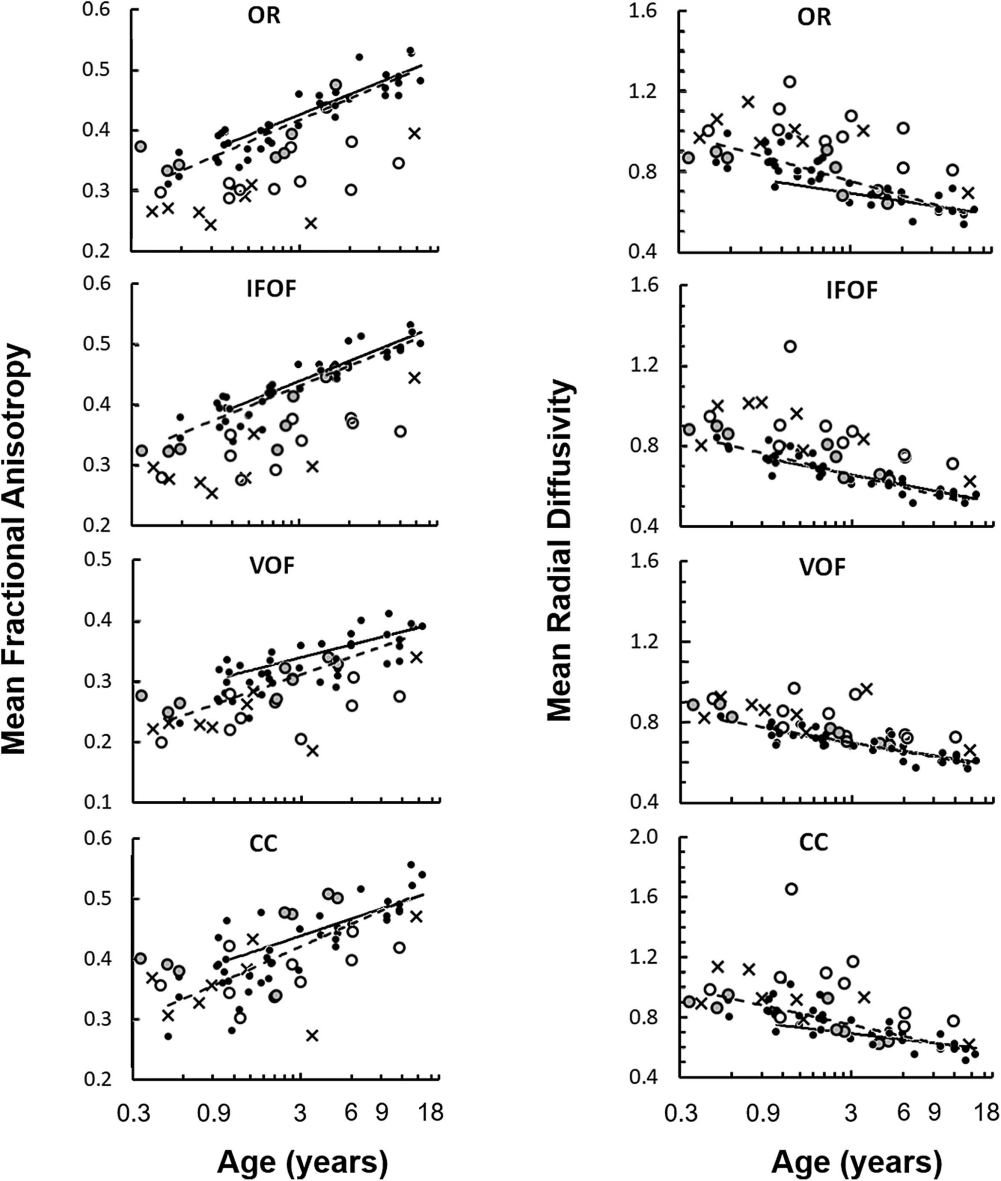
Representative mean fractional anisotropy (*left side*) and mean radial diffusivity (*right side*) plotted with respect to age on a log scale. Controls are plotted as *black circles* (*solid regression lines* are data acquired at 30 directions, whereas *dotted regression lines* are data acquired at 10 directions). Subjects with a normal and abnormal appearing conventional MRI are plotted as *grey* and *open circles*, respectively). Both hemispheres were averaged for each subject. Separate graphs show white matter tracts for the optic radiations (OR), inferior fronto-occipital fasciculus (IFOF), and vertical occipital fasciculus (VOF).

**Figure 6.**
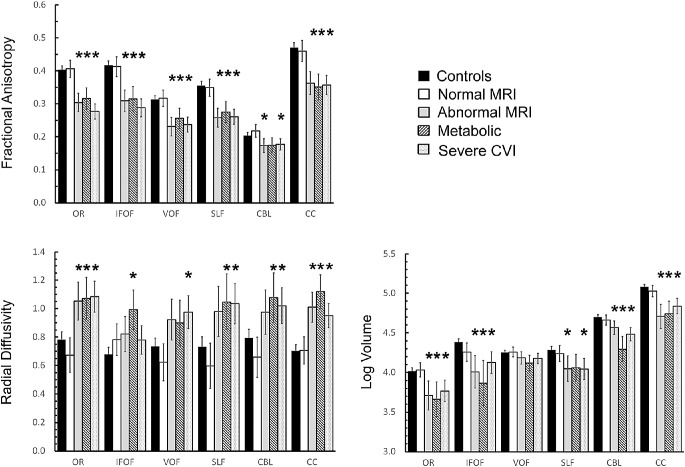
Estimated marginal means of white matter metrics from 8 subjects in the normal MRI group, 6 subjects in the abnormal MRI group, 5 subjects in the metabolic disease group, and 9 subjects in the severe CVI group, and 36 controls of the same age range. The factors age at MRI scan, number of gradient directions, and voxel volume were used as covariates. Data are shown for six different white matter tracts. *Asterisks* denote a significant difference from controls after Bonferroni correction (*P* < 0.01).

Controls had linear relationships of mean FA and RD with log age (Fig. 5), as previously reported.^32^^–^^34^ Subjects showed variable decreased FA and increased RD across multiple tracts (MD data were similar to RD data). The number of gradient directions had a small but detectable effect on mean FA and RD values. We used analysis of covariance (ANCOVA) to compare subjects and controls while accounting for variance associated with number of gradient directions, voxel volume, and age. For ANCOVA, we matched a subset of controls (*n* = 36) to the subjects based on the number of gradient directions, MRI scan date within 6 months, and age range (mean age = subjects 2.8 years, controls = 3.6 years, *P* = 0.3 by *t*-test). We then analyzed the marginal means, which represent averages after adjusting for the effects of number of gradient directions, voxel volume and log age. Figure 6 and Supplementary Figure S2 show subjects with a normal conventional MRI were not different from controls across all white matter tracts for FA, RD, MD, and tract volumes. Subjects in the abnormal MRI, metabolic disease, and severe CVI groups all had evidence of reduced FA, increased RD and MD, and lower volumes across multiple white matter tracts. FA, in particular, showed significantly reduced values for almost all tracts of interest (OR, IFOF, VOF, SLF, CBL, CC, FP, OP, PP, and TP).

Across all subjects, only the OR and OP white matter tracts were significantly correlated with VEP amplitude and SNR (*P* < 0.01, Bonferroni correction). Amplitude was positively correlated with FA (r = 0.83 and 0.81 for OR and OP tracts, respectively) and negatively correlated with RD (r = −0.65 and −0.70 for OR and OP tracts). SNR was positively correlated with FA (r = 0.70 and 0.66 for OR and OP tracts) but not for RD (r = −0.33 and −0.39 for OR and OP tracts). Latency was not correlated with FA, RD, MD, or tract volume.

## Discussion

We describe a group of children with clinical characteristics of CVI with normal VEPs who show absent, or abnormal, oculomotor responses to visual stimuli. Two thirds of these subjects had white matter abnormalities on conventional MRI scans and had abnormal white matter metrics on DTI tractography. Wygnanski-Jaffe et al.^16^ also reported children with CVI having robust VEPs despite appearing “clinically blind.” A subset of their subjects had delayed myelinization or loss of white matter volume. Previously, we reported robust VEPs despite absent visual tracking in infants with CVI due to extensive neuronal migration defects.^17^ Unlike previous studies, our analysis links VEPs, eye movement recording, neuroimaging, and DTI tractography. Collectively the data indicate that a subset of children with CVI have a primary defect in the connectivity and/or transformation of visual information into appropriate oculomotor behaviors.

We examined several white matter tracts originating in visual cortex and included the cerebellum and vermis, which are involved in the calibration of oculomotor tasks. The IFOF provides visual, oculomotor, and attentional connections between occipital cortex (fusiform, lingual, and lateral occipital areas) with areas in the temporal, frontal, and parietal lobes,^35^^–^^37^ and the SLF, which links frontal eye fields with areas of the posterior parietal cortex within and in the vicinity of the IPS.^35^^,^^38^ The OP, PP, and TP tracts are cortico-pontine fibers that connect the occipital, parietal, and temporal cortices to the pons, and likely play a role in eye movements. Subjects with abnormal findings on conventional MRI had global abnormalities in white matter metrics, principally FA, and reduced volume for almost all tracts of interest. The DTI data suggest a combination of factors might play a role such as fiber arrangements, degree of myelination, axonal integrity, or dysregulation. However, a subset of children with visuomotor dysfunction had normal findings on conventional MRI and DTI tractography. These children may have abnormalities of white matter connectivity that are not detectable with current imaging techniques or have other unidentified deficits.

We classified visuomotor dysfunction from CVI on the basis of a normal VEP. Visuomotor dysfunction should not be confused with ocular motor apraxia, Moebius syndrome, optic ataxia,^6^ or delayed visual maturation.^3^^,^^21^ Children with these conditions present with poor visual tracking in infancy but have normal visual acuity, normal VEPs, and eventually develop compensatory eye and head movements, if needed, to orient to visual stimuli. Findings such as strabismus, developmental delay, seizure disorders, gross motor function, and genetic abnormalities did not reliably differentiate subjects with visuomotor dysfunction from those with severe CVI due to loss of visual cortical activation. VEP latency delay was inconsistent across the visuomotor dysfunction subgroups, and VEP latency was not correlated with DTI metrics (unlike findings in demyelinating diseases^39^). One third of the visuomotor dysfunction group had inborn errors of metabolism or abnormal regulation of mitochondrial energy metabolism. The frequency of visuomotor dysfunction is expected to be low,^16^ but our study highlights the importance of recognizing possible multifactorial disorders in a child with presumed CVI that cannot be attributed to prematurity, hypoxic-ischemia or cerebral infarct, hydrocephalus, or a gross defect in cortical migration. The family, rehabilitation specialists, and teachers should be apprised that these children may process visual information despite their lack of oculomotor control. This in turn may help professionals better understand management of the child's complex health and developmental needs.^1^^,^^6^^,^^14^ Further studies will be required to understand how to manage these children and elucidate the etiology of visuomotor dysfunction.

There are several important caveats to this study using retrospective data over variable test periods and limited follow-up duration. DTI acquisition was of clinical quality and might be impacted by development of diffusion within radial glia fibers. Reconstruction of white matter bundles by deterministic tractography is complex and dependent on DWI acquisition parameters (e.g. age, brain volume, the number of gradient directions, accurate seeding, complex crossings of bundles, and streamline parameters). As the VEP is a generalized electrophysiological response, it can be nonspecific to local visual field defects and abnormalities of higher order visual processing in CVI.^1^^,^^6^^,^^7^^,^^20^^,^^40^^–^^46^ This research could not differentiate high-level attention disorders from visuomotor dysfunction, or address long-term outcomes that may lead to clarification of a specific neurological disorder. Another caveat is that our study used pattern VEPs to provide an index of conduction latency in children with white matter disorders. The VEP stimuli were limited to low spatial frequencies that could accommodate the young age, poor fixation, and reduced Teller card acuities in all subjects. We cannot rule out that high frequency stimuli testing in all subjects would detect greater levels of visual cortex dysfunction. Further studies using steady-state or sweep VEPs with age-matched controls can help to elucidate cortical function in visuomotor dysfunction, such as extrapolated grating acuity,^1^^,^^2^^,^^40^ contrast and luminance sensitivity,^41^^,^^42^ motion,^1^^,^^13^ and vernier acuity.^44^^,^^45^ Recent methodologies, such as multifocal VEP, functional MRI, positron emission tomography (PET), and single photon emission computed tomography (SPECT) might further elucidate mechanisms of visuomotor dysfunction in this population, however, performing these tests would be challenging because these subjects will likely require anesthesia followed by careful alignment of the stimulus with the fovea.

## Supplementary Material

Supplement 1

Supplement 2

Supplement 3
