# Supplementary material for: Cerebral Visual Impairment Characterized by Abnormal Visual Orienting Behavior With Preserved Visual Cortical Activation
Source: Invest Ophthalmol Vis Sci. 2021 May 13;62(6):15. doi: 10.1167/iovs.62.6.15 (PMC8132015; doi:10.1167/iovs.62.6.15)

Supplemental Figure 1. Top, Horizontal OKN gain for 2 subjects a normal MRI (circles), 7 subjects with abnormal MRI (triangles), and 2 subjects with metabolic disorder (crosses). Bottom, Vertical OKN gain for the same subjects, except for 3 subjects with abnormal MRI who did not tolerate testing. Data from each eye is plotted, when available. Grey shaded area is total range of controls.

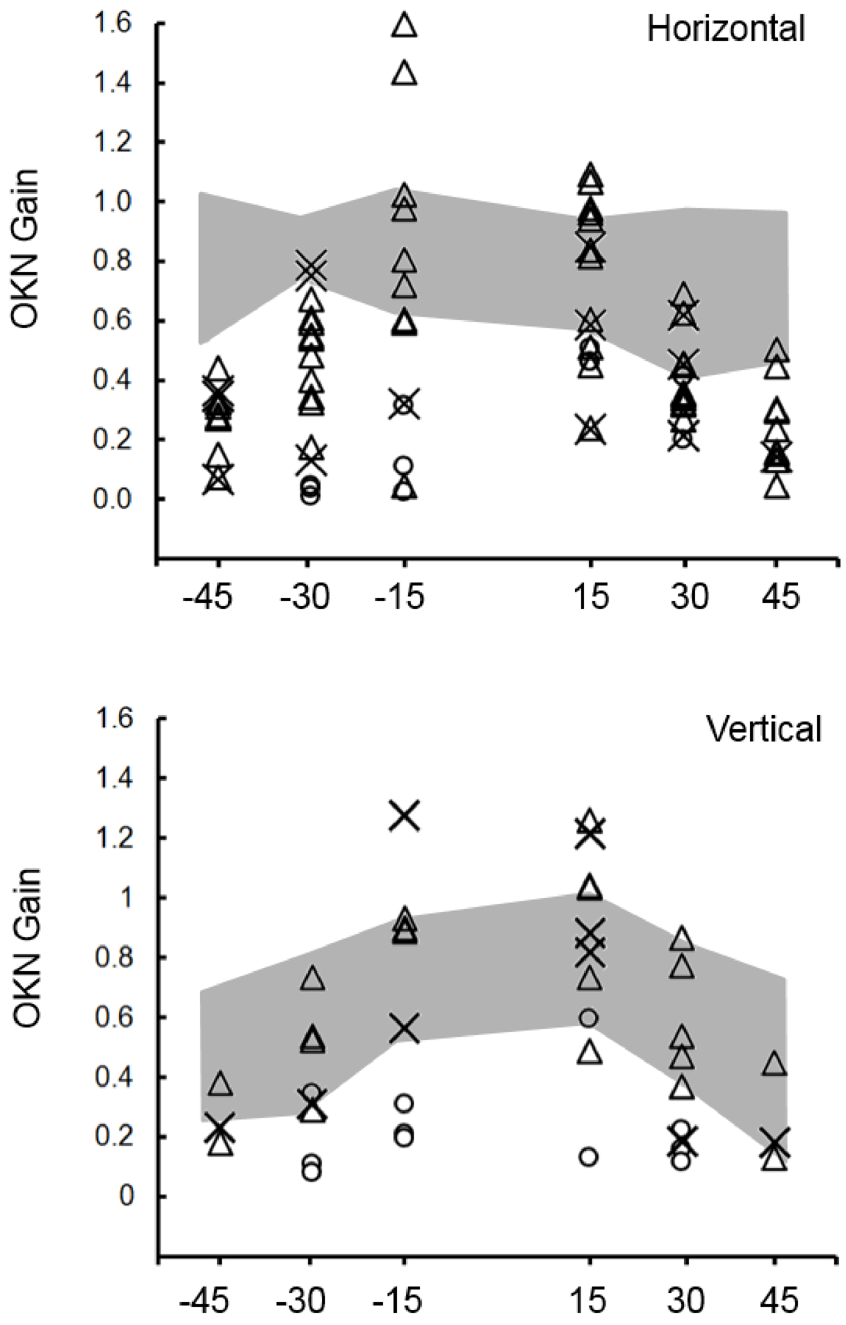

Supplement: Supplement 1 [file iovs-62-6-15_s001.pdf]
