# Supplementary material for: Cerebral Visual Impairment Characterized by Abnormal Visual Orienting Behavior With Preserved Visual Cortical Activation
Source: Invest Ophthalmol Vis Sci. 2021 May 13;62(6):15. doi: 10.1167/iovs.62.6.15 (PMC8132015; doi:10.1167/iovs.62.6.15)

Supplemental figure 2. Estimated marginal means of white matter metrics for cortico-pontine tracts. Conventions are the same as in Figure 6. Asterisks denote a significant difference from controls after Bonferroni correction ($p < 0.01$).

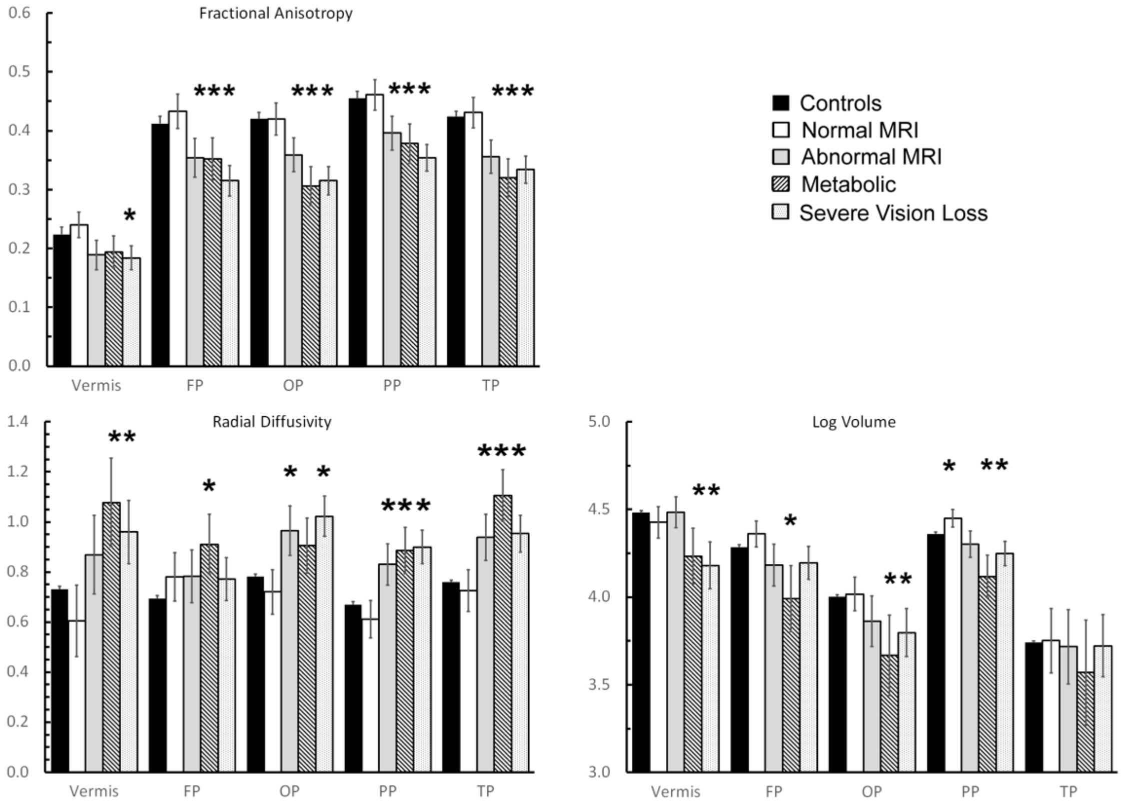

Supplement: Supplement 2 [file iovs-62-6-15_s002.pdf]
