# Supplementary material for: Cerebral Visual Impairment Characterized by Abnormal Visual Orienting Behavior With Preserved Visual Cortical Activation
Source: Invest Ophthalmol Vis Sci. 2021 May 13;62(6):15. doi: 10.1167/iovs.62.6.15 (PMC8132015; doi:10.1167/iovs.62.6.15)
Supplement: Supplement 3 [file iovs-62-6-15_s003.pdf]

Supplemental Table 1. Detailed Subject Data								
Case / Sex	Age first visit	TAC cy/deg	logMAR	F/U Age	TAC cy/deg	logMAR	MRI	Notes
**Normal MRI**								
1 / M	0.25	0.42	0.96	0.40	3.20	0.26	Normal, macrocephaly	na
2 / F	0.58	< 0.23	3.00	1.25	0.31	1.62	Normal	ST3GAL5 variant (c.353delA), ST3GAL5 variant (c.719T>A)†
3 / F	0.92	1.30	0.77	2.25	4.70	0.55	Normal	32 kb del. 11p14.3
4 / F	1.58	3.10	0.59	2.92	4.70	0.62	Normal	SLC6A1 p.Lys36Glu fs*171
5 / M	1.92	2.40	0.80	na			Normal	KCNQ3 c. 1067 C>T p. A356V##
6 / M	2.33	< 0.23	3.00	3.25	1.60	1.10	Normal	Normal SCN1A / CGH array chip
7 / M	2.50	< 0.23	3.00	na			Normal	chromosome 2 q24.3 deletion**
8 / M	3.00	< 0.23	3.00	na			Normal	5.4% homozygosity in SNP array
9 / M	3.08	< 0.23	3.00	4.80	2.40	1.11	Normal	Normal CGH
10 / M	6.00	1.60	1.28	na			Normal	Normal Fragile X,^^
**Abnormal MRI**								
11 / F	0.50	3.10	0.37	1.50	3.10	0.59	WM, CC, DCV	Normal CGH
12 / M	0.60	< 0.23	3.00	1.75	< 0.23	3.00	WM, DCV	Normal CGH
13 / F	0.83	< 0.23	3.00	4.90	0.47	1.90	WM, CC, RM	KCNQ2: c.602G>A, p. Arg 201His (R201H)^
14 / M	0.92	4.70	0.33	2.20	9.80	0.24	WM (occipital), MCM	na
15 / M	0.92	6.50	0.25	na			WM, RM	na
16 / F	1.00	< 0.23	3.00	na			WM, CC, DCV, DCRB	PEX7 c.120C>G (p.Y40X), and c.694C>T (p.R232X)#
17 / F	1.08	< 0.23	3.00	3.10	< 0.23	3.00	WM, CC	na
18 / M	1.08	< 0.23	1.47	2.60	1.60	1.02	CC, borderline DCV, MCM	none found^^
19 / F	1.35	< 0.23	3.00	6.25	14.00	0.30	DCRB/ hypoplasia	normal karotype
20 / F	1.42	2.40	0.68	8.90	3.10	0.98	WM, CC, MCM, partial RM	DDX3X (c.1439G>C)+
21 / M	1.67	0.42	1.51	na			WM, CC, DCV	DNM1:p.Ser175Phe and DNM1:p.Ala177Pro*
22 / M	1.80	0.47	1.40	1.50	0.47	1.52	WM, CC, mild DCV, partial RM	na
23 / F	2.17	< 0.23	3.00	na			WM, CC	none found^^
24 / F	5.00	< 0.23	3.00	11.10	0.23	2.13	DCV, PVNH	Xq11 del 679 kb
**Metabolic disorder**								
25 / F	0.58	< 0.23	3.00	7.80	4.70	0.81	WM, Lac, RM	PDHX c.211G>T (p.G71X) and c.850C>T (p.R284X)++
26 / F	0.83	0.42	1.40	1.33	0.31	1.62	progressive WM, DCV (sparing occipital lobes and cerebellum)	probable mitochondrial DNA depletion
27 / M	0.83	< 0.23	3.00	0.83	< 0.23	3.00	DCRB	glutathione peroxidase deficiency, none found^^

28 / M	0.92	1.60	0.59	8.80	3.10	0.98	WM, CC, DCV, RM	complex I, IV deficiency
29 / F	1.75	9.80	0.19	na			WM, CC, T2 signal midbrain	elevated lactate and pyruvate, normal karotype
30 / F	10.00	6.50	0.42	10.00	< 0.23	3.00	CC, DCRB, hypoplasia of vermis	mitochondrial cytopathy (complex II/III and I/III), none found^^
**Severe Vision loss**								
31 / F	0.42	< 0.23	3.00	na			Diffuse WM, NAA	ASPA c.79 G>A, p.G27R^^^
32 / M	0.50	1.30	0.77	5.75	1.30	1.37	periventricular calcifications	CMV
33 / F	0.58	0.31	1.37	na			diffuse HII	GCH1 (1 c.149delC) +++
34 / F	0.92	< 0.23	3.00	2.10	6.50	0.41	PVNH, partial agenesis SP	chromosome 5p dup
35 / F	1.00	< 0.23	3.00	na			DCV, CC, DCRB, brainstem atrophy	none found^^
36 / F	1.08	< 0.23	3.00	3.50	< 0.23	3.00	Progressive WM, CC, DCV, MCM	complex I, II, III, IV deficiency
37 / F	1.33	< 0.23	3.00	3.80	< 0.23	3.00	WM, CC	chromosome 3p26.3 dup
38 / M	1.50	< 0.23	3.00	na			WM, DCRB	complex I, III, IV deficiency
39 / F	1.92	< 0.23	3.00	3.40	1.30	1.24	WM, CC, DCV, Lac	UBA5 p.Gly267*/p.Ala371Thr)^^^^
40 / F	2.25	< 0.23	3.00	3.80	< 0.23	3.00	DCV, agenesis CC	PDHA1 frameshift c.1048_1054delAGTAAGA (p.Ser350Valfs)***

Age is in years, F/U = last follow-up, visual acuity by Teller cards (TAC) reported in cycles/degree (cy/deg) and also converted to age corrected log minimum angle of resolution (logMAR). WM = delayed or abnormal white matter signal, CC = thin corpus callosum, DCV = decreased cerebral volume, DCRB = decreased cerebellar volume, HII = hypoxic ischemic injury, MCM = mega-cisterna magna, PVNH = periventricular nodular heterotopia, Lac = elevated lactate peak, NAA = elevated N-acetylaspartate peak, SP = septum pellucidum, RM = recovered age-appropriate myelination, na = not available. Children with their initial ophthalmology visit after 1 year of age were referred to our service for specialized testing.

*both variants are in cis. p.Ala177Pro is a pathological variant [Von Spiccak et al., 2017]

^likely pathological variant [Goto et al., 2019]

#Biallelic truncating mutations giving rise to rhizomelic chondrodysplasia punctate [Wanders and Waterham, 2005].

+ De novo variant likely pathological variant in X-linked Toriello-Carey syndrome.

** The deletion in this region includes SCN1A, gives rise to Dravet syndrome [Harkin et al., 2007]

^^No pathological variants found on infant epilepsy panel or whole exome sequencing

De novo variant-likely pathological

++ PDHX also known as PDX1, pathological biallelic premature stop sequences [Brown et al., 2006]

†De novo ST3GAL5 variant, both mutations likely pathological

^^^Canavan disease

+++ pathogenic mutation for dopa-responsive dystonia

CMV, positive congenital cytomegalovirus titers

^^^^ Frameshift mutation not reported; the missense mutation produces a hypomorphic change [Arnadottir et al., 2017]

von Spiczak S, Helbig KL, Shinde DN, et al. Epi4K Consortium; EuroEPINOMICS-RES NLES Working Group. DNM1 encephalopathy: A new disease of vesicle fission. Neurology. 2017 Jul 25;89(4):385-394.

Goto A, Ishii A, Shibata M, et al. Characteristics of KCNQ2 variants causing either benign neonatal epilepsy or developmental and epileptic encephalopathy. Epilepsia. 2019 Aug 16. doi: 10.1111/epi.16314. [Epub ahead of print], PMID: 31418850

Wanders RJ, Waterham HR. Peroxisomal disorders I: biochemistry and genetics of peroxisome biogenesis disorders. Clin Genet. 2005 Feb;67(2):107-33.

Harkin LA, McMahon JM, Iona X, Dibbens L, Pelekanos JT, Zuberi SM, et al. A; Infantile Epileptic Encephalopathy Referral Consortium, Sutherland G, Berkovic SF, Mulley JC, Scheffer IE. The spectrum of SCN1A-related infantile epileptic encephalopathies. Brain. 2007 Mar;130(Pt 3):843-52.

Brown RM, Head RA, Morris AA, et al. Pyruvate dehydrogenase E3 binding protein (protein X) deficiency. Dev Med Child Neurol. 2006 Sep;48(9):756-60.

Arnadottir GA, Jensson BO, Marelsson SE, et al. Compound heterozygous mutations in UBA5 causing early-onset epileptic encephalopathy in two sisters. BMC Med Genet. 2017 Oct 2;18(1):103. doi: 10.1186/s12881-017-0466-8.
